# Gfi1-Mediated Repression of *c-Fos*, *Egr-1* and *Egr-2*, and Inhibition of ERK1/2 Signaling Contribute to the Role of Gfi1 in Granulopoiesis

**DOI:** 10.1038/s41598-018-37402-z

**Published:** 2019-01-24

**Authors:** Yangyang Zhang, Nan Hu, Fan Dong

**Affiliations:** Department of Biological Sciences, University of Toledo, Toledo, Ohio 43606 USA

## Abstract

Gfi1 supports neutrophil development at the expense of monopoiesis, but the underlying molecular mechanism is incompletely understood. We recently showed that the G-CSFR Y729F mutant, in which tyrosine 729 was mutated to phenylalanine, promoted monocyte rather than neutrophil development in myeloid precursors, which was associated with prolonged activation of Erk1/2 and enhanced activation of c-Fos and Egr-1. We show here that Gfi1 inhibited the expression of c-Fos, Egr-1 and Egr-2, and rescued neutrophil development in cells expressing G-CSFR Y729F. Gfi1 directly bound to and repressed *c-Fos* and *Egr-1*, as has been shown for *Egr-2*, all of which are the immediate early genes (IEGs) of the Erk1/2 pathway. Interestingly, G-CSF- and M-CSF-stimulated activation of Erk1/2 was augmented in lineage-negative (Lin^−^) bone marrow (BM) cells from *Gfi1*^−/−^ mice. Suppression of Erk1/2 signaling resulted in diminished expression of c-Fos, Egr-1 and Egr-2, and partially rescued the neutrophil development of *Gfi1*^−/−^ BM cells, which are intrinsically defective for neutrophil development. Together, our data indicate that Gfi1 inhibits the expression of c-Fos, Egr-1 and Egr-2 through direct transcriptional repression and indirect inhibition of Erk1/2 signaling, and that Gfi1-mediated downregulation of c-Fos, Egr-1 and Egr-2 may contribute to the role of Gfi1 in granulopoiesis.

## Introduction

Gfi1 plays a critical role in hematopoiesis^1–3^. *Gfi1* ablation in mice impaired the self-renewal ability of hematopoietic stem cells (HSCs)^4–6^, indicating that Gfi1 is required for the functional integrity of HSCs. *Gfi1*^−/−^ mice also show defects in B and T-cell development. The most significant phenotype of *Gfi1*^−/−^ mice is a lack of mature neutrophils accompanied by an expansion of granulocyte-monocyte progenitors (GMPs) and immature monocyte-like myeloid cells^7–10^. Myeloid precursors from *Gfi1*^−/−^ mice are unable to differentiate into mature neutrophils *in vitro*, but instead give rise to atypical monocytes. In contrast, overexpression of Gfi1 accelerates neutrophilic differentiation at the expense of monocyte formation^11,12^. Thus, Gfi1 promotes the neutrophil development and antagonize the alternative monocyte/macrophage fate. Consistent with its role in granulopoiesis, mutations in *GFI1* have been identified in patients with severe congenital neutropenia (SCN)^3,13,14^, a condition characterized by a profound absolute neutropenia occurring in early life and a maturation arrest of myeloid precursors in the bone marrow. Expression of an SCN-derived dominant negative (DN) Gfi1 mutant, N382S, in mouse Lin^−^ BM cells led to monocyte development in response to G-CSF at the expense of granulopoiesis^11^.

The molecular mechanism by which Gfi1 favors neutrophil over monocyte development is incompletely understood.Gfi1 has been shown to repress *PU*.*1*, *Egr-2* and *CSF1* encoding M-CSF^11,15,16^, which promotes monocyte development. Notably, targeted deletion of *Csf1* rescued granulopoiesis that was blocked by the Gfi1 N382S mutant in mouse Lin^−^ BM cells^11^, suggesting that repression of *Csf1* represents an important mechanism by which Gfi1 supports granulopoiesis. In addition, Gfi1 has been shown to repress miR21 and miR-196b, which may also contribute to its role in resolving neutrophil versus monocyte fate^17^.

We recently demonstrated that G-CSF and M-CSF instruct neutrophil and monocyte fates, respectively, in part through differential activation of Erk1/2 and the downstream targets c-Fos and Egr-1^18^. Specifically, M-CSF stimulates strong and prolonged activation of Erk1/2-c-Fos/Egr1 signaling pathway in comparison with the weak and transient activation of the pathway in response to G-CSF. Here we show that Gfi1 directly represses *c-Fos*, *Egr-1* and *Egr-2*, and negatively regulates Erk1/2 activation. We further demonstrate that inhibition of Erk1/2 signaling led to diminished expression of c-Fos, Egr-1 and Egr-2, and partially rescued the neutrophil development in Lin^−^ BM cells from *Gfi1*^−/−^ mice.

## Results

### Gfi1 rescues neutrophil development in 32D/Y729F and FDCP/Y729F cells independent of M-CSF signaling

**Figure 1:**
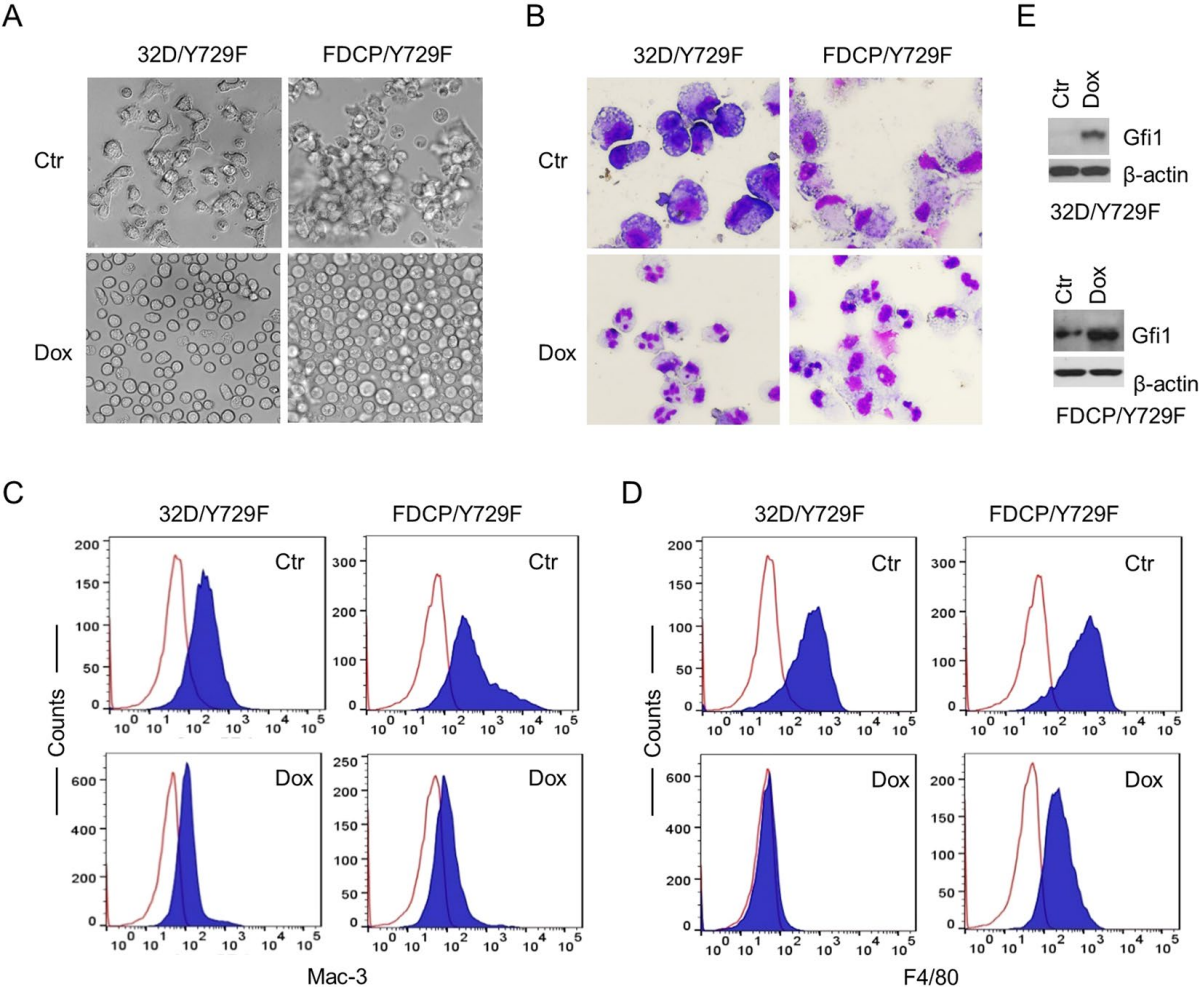
Gfi1 overexpression rescues neutrophil development. 32D/Y729F/Gfi1 and FDCP/Y729F/Gfi1 cells were cultured in G-CSF in the absence (Ctr) or presence of Dox. (**A**) Cell growth behaviors were examined on day 6 for 32D/Y729F/Gfi1 cells and day 2 for FDCP/Y729F/Gfi1 cells. (**B**) Cell morphology was evaluated on day 6 for 32DY729F/Gfi1 cells, and on day 3 and day 5 for FDCP/Y729F/Gfi1 cells in the absence and presence of Dox, respectively (May Grünwald Giemsa staining). The expression of Mac-3 (**C**) and F4/80 (**D**) was examined on day 3. (**E**) Gfi1 expression following Dox induction for 24 hours was examined by Western blot analysis.

We recently showed that murine myeloid 32D and multipotent FDCP-mix A4 cells expressing the human G-CSFR Y729F (32D/Y729F and FDCP/Y729F), in which tyrosine 729 in the cytoplasmic domain was mutated to phenylalanine, underwent monocyte rather than neutrophil development in response to G-CSF^18^. Parental 32D and FDCP-mix A4 cells expressed no detectable M-CSF receptor and showed no response to M-CSF (data not shown). To address whether Gfi1 suppressed monopoiesis independent of *M-CSF* signaling, we transduced 32D/Y729F and FDCP/Y729F with the lentiviral pPMPrtTA-Gfi1-IRES-GFP construct in which Gfi1 expression was driven by the tetracycline-response element (TRE)^19^. The transduced cells (32D/Y729F/Gfi1 and FDCP/Y729F/Gfi1) were cultured in G-CSF-containing medium without or with doxycycline (Dox). In the absence of Dox, 32D/Y729F/Gfi1 and FDCP/Y729F/Gfi1 cells displayed features typical of monocyte development, including increased cell sizes, adherence to culture dishes, monocyte/macrophage morphology, and increased expression of macrophage surface marker F4/80 (Fig. 1). Dox induction of Gfi1 expression completely restored G-CSF-induced neutrophil development in both cell types, which was associated with upregulation of neutrophil differentiation markers neutrophil elastase (NE) and lactoferrin (LF), and downregulation of monocyte/macrophage differentiation markers M-CSF and MMP-12 (Suppl. Fig. 1). Induction of Gfi1 expression alone did not lead to neutrophil development in the two cell lines cultured in IL-3. Thus, Gfi1 may promote neutrophil fate independent of its inhibitory effect on M-CSF expression.

### Gfi1 represses *c-Fos* and *Egr-1*

**Figure 2:**
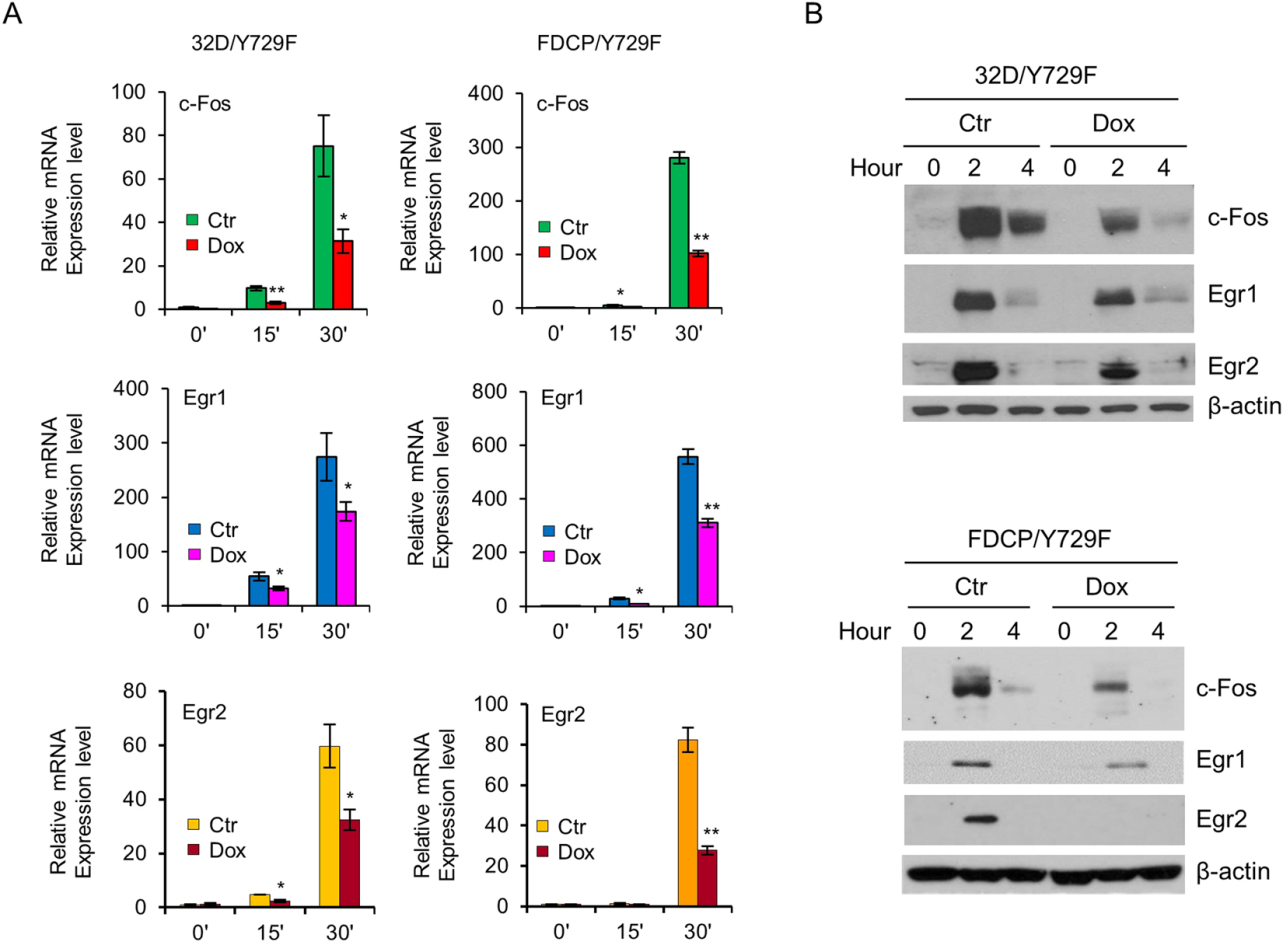
Gfi1 suppresses the expression of c-Fos, Egr-1 and Egr-2. (**A**) 32D/Y729F/Gfi1 and FDCP/Y729F/Gfi1 cells were cultured in the absence (Ctr) or presence of Dox for 12 hours. Cells were then starved for 2 hours and stimulated with G-CSF for the indicated times prior to evaluation of c-Fos, Egr-1 and Egr-2 mRNA (**A**) and protein levels (**B**).

**Figure 3:**
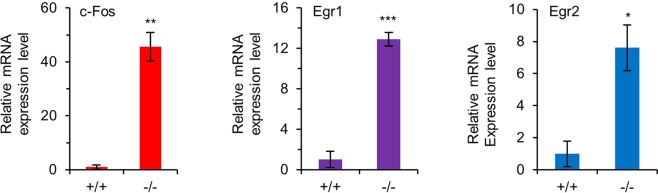
The expression of c-Fos, Egr-1 and Egr-2 mRNAs is markedly increased in Lin^−^ BM cells from Gfi1^−/−^ mice. Cells were isolated from 6–8 week old Gfi1^+/+^ and Gfi1^−/−^ mice. The mRNA levels of c-Fos, Egr-1 and Egr-2 were assessed by qRT-PCR.

Monocyte development mediated by G-CSFR Y729F was associated with prolonged activation of Erk1/2 and subsequently augmented activation of *c-Fos* and *Egr-1*, and knockdown of c-Fos or Egr-1 in 32D/Y729 and FDCP/Y729F cells rescued neutrophil development^18^. Notably, Gfi1 has been shown to repress *Egr-2* and downregulate Egr-1 expression^20^. We examined whether Gfi1 had an effect on the expression of c-Fos and Egr-1 in response to G-CSF. The mRNAs and protein levels of c-Fos and Egr-1 were rapidly induced following G-CSF stimulation, but their induction was markedly attenuated in Dox-treated 32D/Y729F/Gfi1 and FDCP/Y729F/Gfi1 cells (Fig. 2). Consistent with previous report^20^, Gfi1 repressed Egr-2 expression. We further investigated whether Gfi1 regulated the expression of c-Fos and Egr-1 in primary BM cells. As shown in Fig. 3, the mRNA levels of c-Fos and Egr-1 were considerably higher in Lin^−^ BM cells from *Gfi1*^−/−^ mice than in cells from *Gfi1*^+/+^ mice. As expected, Egr-2 expression was also increased in *Gfi1*^−/−^ BM cells.

**Figure 4:**
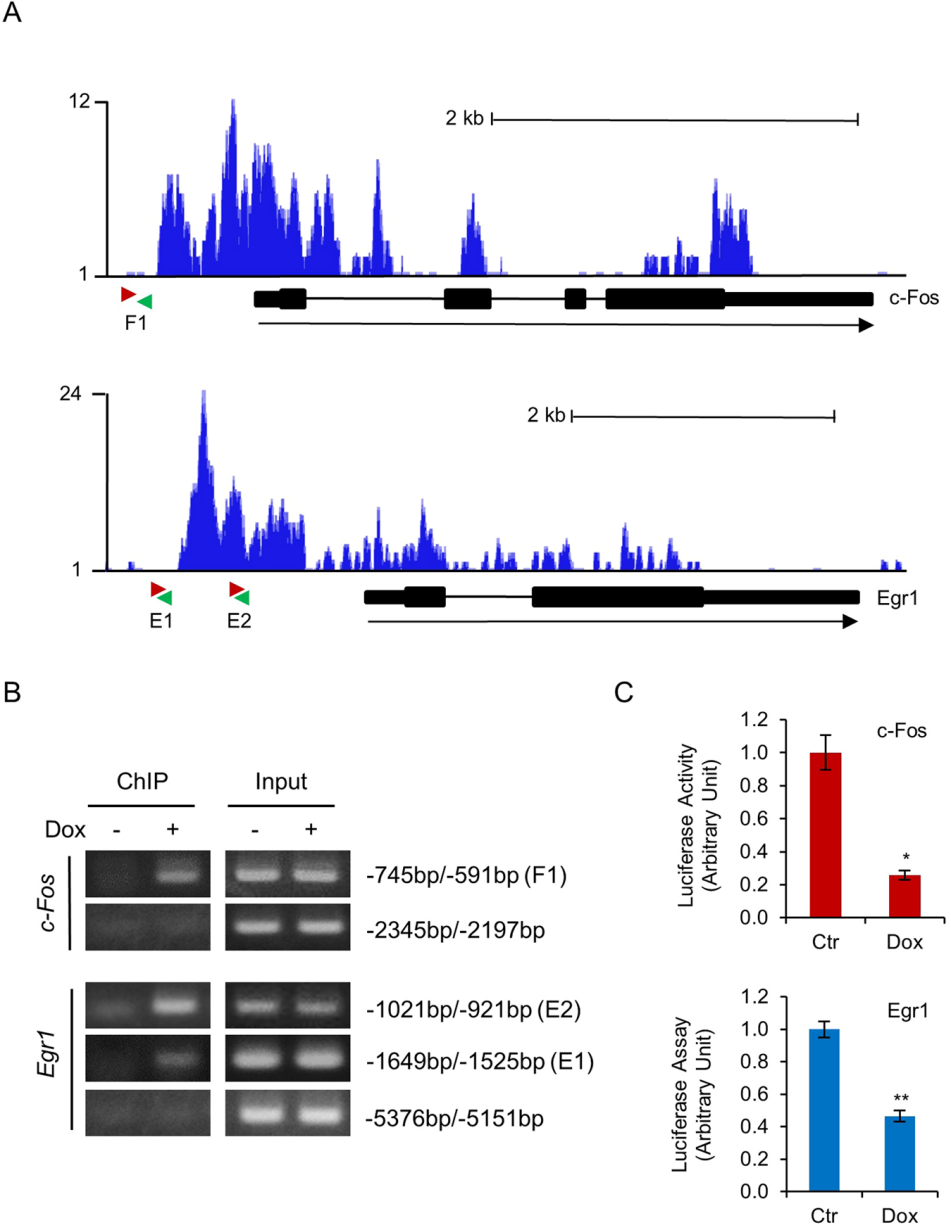
Gfi1 represses *c-Fos* and *Egr-1* through direct binding to their promoters. (**A**) Gfi1 binding patterns at the *c-Fos* (upper panel) and *Egr-1* (lower panel) based on the ChIP-seq data submitted by Möröy’s research group^21^. The pink and green arrows denote the forward and reverse primers, respectively, used to amplify the promoter regions at c-Fos (F1) and Egr-1 (E1 and E2) loci. (**B**) 32D/Y729F/Gfi1 cells were cultured with or without Dox for 24 hours. ChIP assays were carried out using the anti-mouse Gfi1 antibody. The indicated regions of *c-Fos* and *Egr-1* promoters were amplified by PCR. (**C**) 32D/Y729F/Gfi1 cells were transfected with pGL3-basic vector containing *c-Fos* or *Egr-1* promoter fragment and cultured in G-CSF with or without Dox. Luciferase activities were measured 24 hours later.

Gfi1 has been shown to bind to *Egr-2*^20^. Analysis of the ChIP-seq data (GSE31657) submitted by Möröy’s research group^21^ indicated that Gfi1 bound to the promoters of *c-Fos* and *Egr-1* in murine hematopoietic progenitor cells (Fig. 4A). Examination of *c-Fos* and *Egr-1* promoters using the online transcription factor prediction tool TFBIND (http://tfbind.hgc.jp/) revealed potential Gfi1 binding sites at approximate nucleotide positions −786 and −661 of *c-Fos*, and at −1614 and −997 of *Egr-1*. ChIP assays demonstrate that Gfi1 indeed bound to these sites, but not the upstream promoter regions of *c-Fos* and *Egr1* in 32D/Y729F/Gfi1 cells (Fig. 4B). We further performed luciferase reporter assays to determine whether Gfi1 repressed the *c-Fos* and *Egr-1* promoters. As shown in Fig. 4C, Gfi1 repressed the activities of murine *c-Fos* promoter fragment spanning from −1070 to + 30 bp and *Egr-1* promoter fragment spanning from −1780 to + 50 bp in 32DGR/Y729F/Gfi1 cells. As reported in the previous study^20^, Gfi1 also repressed the *Egr-2* promoter (data not shown). Together, these data revealed that *c-Fos* and *Egr-1* are the novel target genes of Gfi1.

### Enhanced activation of Erk1/2 contributes to increased expression of c-Fos, Egr-1 and Egr-2 in Lin^−^ BM cells from *Gfi1*^−/−^ mice

**Figure 5:**
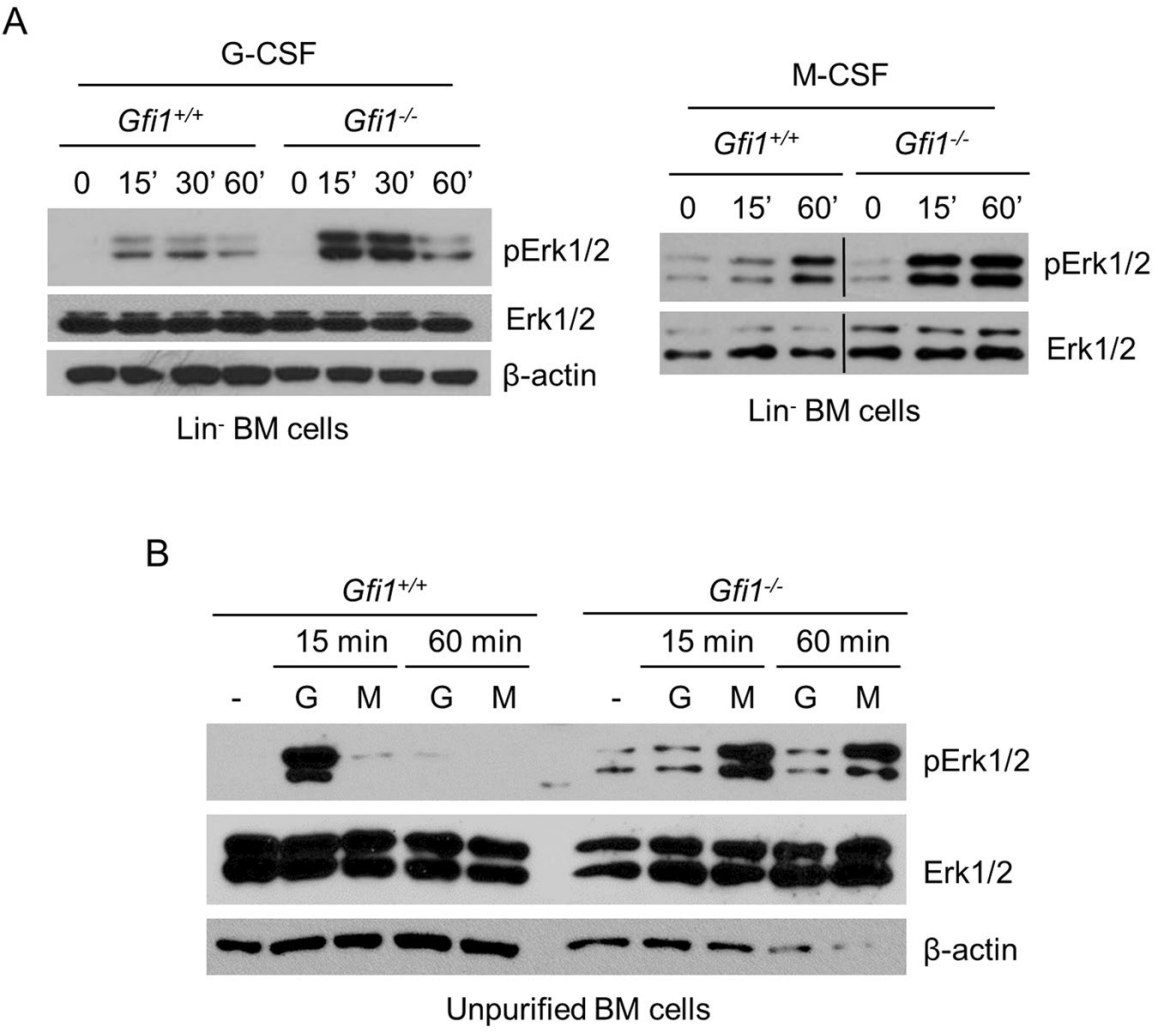
G-CSF- and M-CSF-induced activation of Erk1/2 in different populations of BM cells from *Gfi1*^+/+^ and *Gfi1*^−/−^ mice. Lin^−^ (**A**) and unpurified (**B**) BM cells were isolated from G*fi1*^+/+^ and *Gfi1*^−/−^ mice, and treated with G-CSF (G) or M-CSF (M) for the indicated times. Erk1/2 phosphorylation was examined by Western blot analysis.

It has been shown that *c-Fos*, *Egr-1* and *Egr-2* are the IEGs of the Erk1/2 signaling pathway^22,23^. A previous study showed that G-CSF-stimulated activation of Erk1/2 was significantly reduced in unpurified BM cells from *Gfi1*^−/−^ mice^12^. However, *Gfi1*^−/−^ mice lack mature neutrophils accompanied by an expansion of atypical monocytes in BM and peripheral blood. We therefore examined Erk1/2 activation in Lin^−^ BM cells. Unexpectedly, Erk1/2 activation in response to G-CSF and M-CSF was stronger in *Gfi1*^−/−^ cells than in *Gfi1*^+/+^ cells (Fig. 5A). Notably, when unpurified BM cells were used, G-CSF-stimulated activation of Erk1/2 was strong in *Gfi1*^+/+^ cells, but extremely weak or barely activated in *Gfi1*^−/−^ cells (Fig. 5B), in line with the previous study^12^. In contrast, M-CSF stimulation led to strong Erk1/2 activation in *Gfi1*^−/−^ cells, but not in *Gfi1*^+/+^ cells. Flow cytometric analyses revealed that unpurified BM cells from *Gfi1*^+/+^ mice abundantly expressed G-CSFR, but only a small percentage of these cells weakly expressed M-CSFR whereas unpurified *Gfi1*^−/−^ cells expressed high levels of M-CSFR with minimal expression of G-CSFR (Suppl. Fig. 2). The expression levels of G-CSFR and M-CSFR in the Lin^−^ cells from *Gfi1*^+/+^ and *Gfi1*^−/−^ mice were relatively comparable. Together, these results indicate that Gfi1 negatively regulates Erk1/2 activation in Lin^−^ BM cells whereas the differential activation of Erk1/2 in response to G-CSF and M-CSF in the unpurified BM cells from *Gfi1*^+/+^ and *Gfi1*^−/−^ mice may largely result from the differential expression of G-CSFR and M-CSFR.

**Figure 6:**
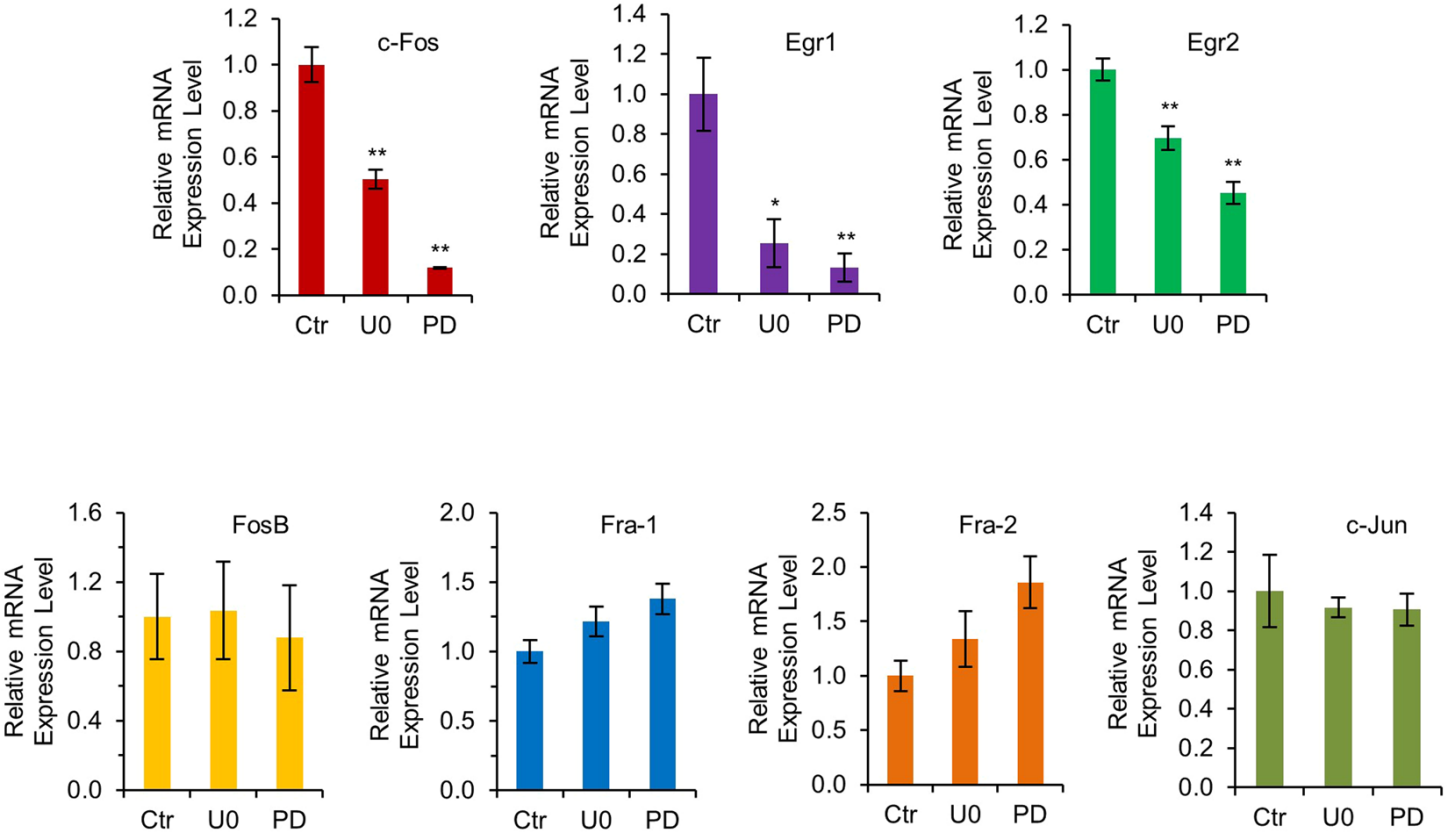
Mek1/2 inhibitors specifically inhibit the expression of Erk1/2 downstream targets c-Fos, Egr-1 and Egr-2. Lin^−^ BM cells from *Gfi1*^−/−^ mice were treated without (Ctr) or with Mek1/2 inhibitors U0126 (UO) or PD0325901 (PD) for 8 hours. The mRNA levels of indicated genes were assessed by qRT-PCR.

The above results suggest that both loss of Gfi1-mediated transcriptional repression and the augmented activation of Erk1/2 may contribute to the increased expression of *c-Fos*, *Egr-1* and Egr-2 in the Lin^−^ BM cells from *Gfi1*^−/−^ mice. We therefore addressed whether inhibition of Erk1/2 signaling diminished the expression of c-Fos, Egr-1 and Egr-2 in *Gfi1*^−/−^ BM cells. As shown in Fig. 6, treatment of *Gfi1*^−/−^ Lin^−^ BM cells with the specific Mek1/2 inhibitors U0126 or PD0325901 resulted in significantly reduced mRNA levels of *c-Fos*, *Egr-1* and *Egr-2*, but had no effect on the expression of other Fos family members, including *FosB*, *Fra-1* and *Fra-2*, and *c-Jun*.

### Mek1/2 inhibitors partially rescue neutrophil development of Lin^−^ BM cells from *Gfi1*^−/−^ mice

**Figure 7:**
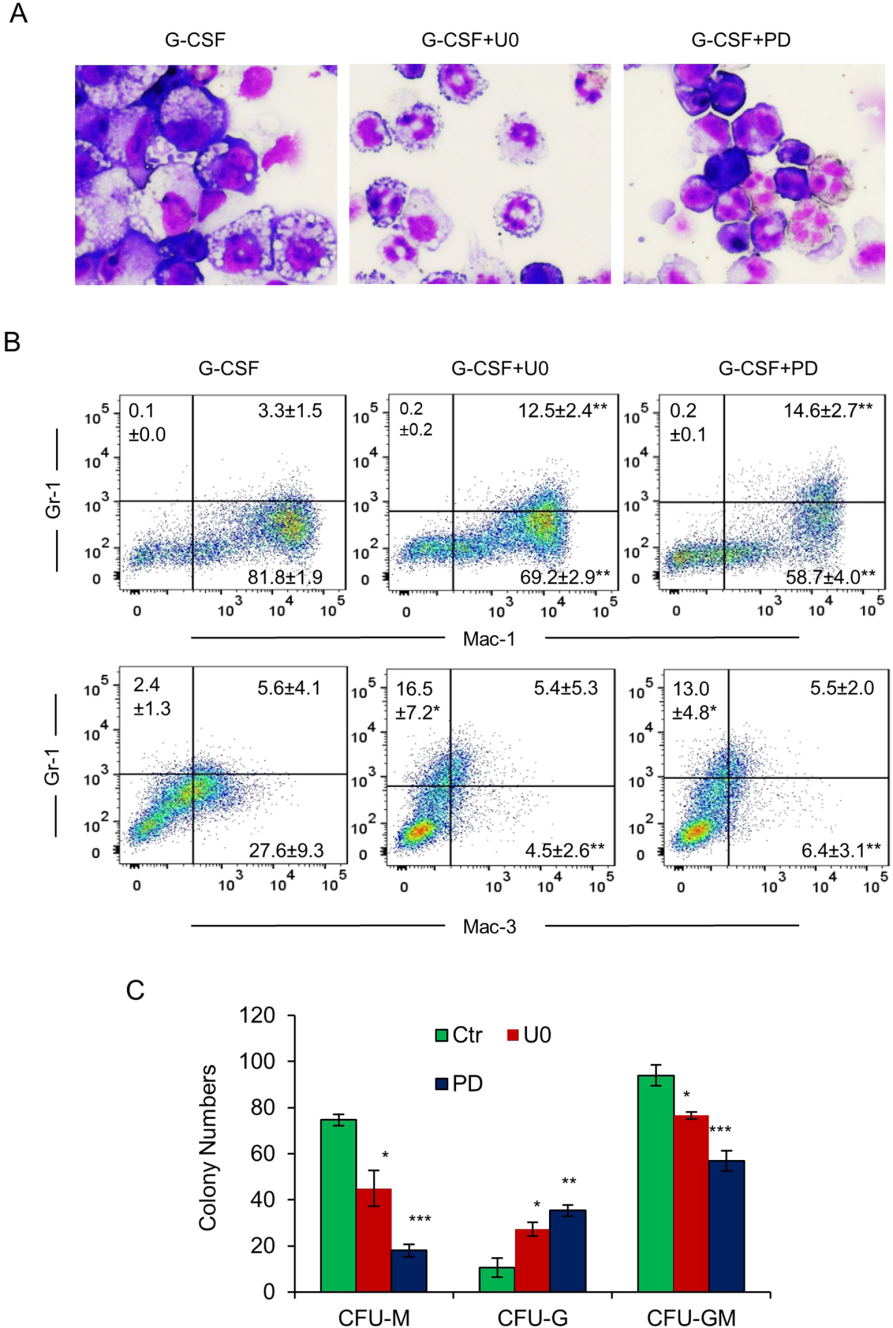
Mek1/2 inhibitors partially rescue neutrophil development in BM cells from Gfi1^−/−^ mice. (**A**) Lin^−^ cells from *Gfi1*^−/−^ mice were cultured in G-CSF-containing medium without or with U0126 (U0) or PD0325901 (PD). Cell morphology was examined on day 3 (May Grünwald Giemsa staining). (**B**) Expression of Gr-1, Mac-1 and Mac-3 was analyzed by flow cytometry on day 3. Data are presented as percentage of positively stained cells (n = 3; mean ± SD). (**C**) BM cells from *Gfi1*^−/−^ mice were cultured in methylcellulose medium containing IL-3, IL-6, SCF and G-CSF without or with U0 or PD. Colonies were counted 7 days later.

BM myeloid precursors from *Gfi1*^−/−^ mice are unable to differentiate into mature neutrophils *in vitro*, but give rise to atypical monocytes/macrophages^8,11^. Consistent with the previous reports, *Gfi1*^−/−^ Lin^−^ BM cells developed into cells with an appearance reminiscent of monocytes/macrophages when cultured in G-CSF (Fig. 7). Because suppression of Erk1/2 signaling in *Gfi1*^−/−^ Lin^−^ BM cells reduced the expression of c-Fos, Egr-1 and Egr-2, we asked whether the Mek1/2 inhibitors rescued neutrophil development of *Gfi1*^−/−^ BM cells. Interestingly, treatment of *Gfi1*^−/−^ BM cells with U0126 or PD0325901 led to a significant shift towards neutrophil development, as evident from the neutrophil-like morphology (Fig. 7A) and reduced percentages of Gr-1^-^/Mac-1^+^ and Gr-1^-^/Mac-3^+^ monocytes with concomitant increase of Gr-1^+^/Mac-1^+^ cells and Gr-1^+^/Mac-3^-^ neutrophils (Fig. 7B). Treatment with U0126 or PD0325901 also upregulated the expression of NE, LF and myeloperoxidase (MPO), but downregulated the expression of M-CSF and MMP12 (Suppl. Fig. 3). In methylcellulose colony formation assays, both U0126 and PD0325901 increased the numbers of CFU-G, but reduced the formation of CFU-M (Fig. 7C). Taken together, these data indicated that inhibition of Erk1/2 signaling resulted in partial restoration of neutrophil development in *Gfi1*^−/−^ BM cells, presumably through suppressing the expression of c-Fos, Egr-1 and Egr-2.

## Discussion

Gfi1 supports the neutrophil development and antagonizes the alternative monocyte/macrophage fate. The molecular mechanism by which Gfi1 favors neutrophil over monocyte development is incompletely understood, but may involve Gfi1-mediated repression of *Pu*.*1*, *Egr-2* and *Csf1* as well as *miR-21* and *miR-196b*^17,24^. It appears that Gfi1-mediated repression of *Csf1* is important for its role in granulopoiesis as *Csf1* ablation rescued granulopoiesis that was blocked by the DN Gfi1 N382S mutant in mouse BM cells^11^. In this paper, we have shown that Gfi1 promotes granulopoiesis independent of its effect on M-CSF signaling. We have further shown that Gfi1 binds to and represses *c-Fos* and *Egr-1*. These data indicate that *c-Fos* and *Egr-1*, along with the previously identified *Egr-2*^20^, are Gfi1 target genes. c-Fos forms the AP-1 protein through heterodimerization with c-Jun. As c-Fos/AP1, Egr-1 and Egr-2 have been shown to promote monopoiesis^25–29^, it is likely that the effects of Gfi1 on neutrophil versus monocyte development are mediated in part through repression of *c-Fos*, *Egr-1* and *Egr-2*.

We have also shown that *Gfi1* ablation results in enhanced activation of Erk1/2 in response to G-CSF and M-CSF in mouse Lin^−^ BM cells, indicating that Gfi1 negatively regulates Erk1/2 signaling. As *c-Fos*, *Egr-1* and *Egr-2* are the IEGs of the Erk1/2 signaling pathway^22,23^, this raises the possibility that Gfi1 may downregulate the expression of c-Fos, Egr-1 and Egr-2 in part through suppression of cytokine-induced activation of Erk1/2 signaling. Indeed, the augmented expression of c-Fos, Egr-1 and Egr-2 in the Lin^−^ BM cells from *Gfi1*^−/−^ cells mice was significantly attenuated upon suppression of Erk1/2 signaling using the Mek1/2 inhibitors. However, it remains to be determined how Gfi1 inhibits Erk1/2 signaling. As Gfi1 is a nuclear protein that functions as a transcriptional repressor, the effect of Gfi1 on Erk1/2 signaling is likely indirect. It is possible that Gfi1 may repress a positive regulator of the Erk1/2 pathway or indirectly increase the expression of a negative regulator of Erk1/2 signaling.

Mutations in *GFI1* have been associated with SCN^3,13,14^. When expressed in mouse BM cells, the SCN-derived DN GFI1 mutant supported monopoiesis, but blocked neutrophil development in response to G-CSF^11^. It has been shown that *Gfi1*^−/−^ myeloid precursors are intrinsically defective for neutrophil development and *in vivo* administration of G-CSF had no effect on neutropenia in *Gfi1*^−/−^ mice^7–9^. In this aspect, it is noteworthy that the MEK1/2 inhibitors U0126 and PD0325901 partially rescued G-CSF-induced neutrophil development in *Gfi1*^−/−^ BM cells, likely through downregulation of the expression of c-Fos, Egr-1 and Egr-2. It would be interesting to explore whether *in vivo* administration of Mek1/2 inhibitors alleviates neutropenia in *Gfi1*^−/−^ mice; if it does, suppression of Erk1/2 signaling could represent a novel therapeutic approach in the treatment of SCN patients with *GFI1* mutations.

## Materials and Methods

### Cell lines and cell culture

Murine myeloid 32D cells and multipotential FDCP-mix A4 cells expressing the different forms of G-CSFR have been described^18,30,31^. 32D and FDCP-mix cells were cultured as described^18^. Briefly, 32D cells were maintained in RPMI-1640 with 10% heat inactivated fetal bovine serum (HI-FBS), 10% WEHI-3B cell-conditioned media as a crude source of murine interleukin-3, and 1% penicillin/streptomycin (P/S). FDCP-mix A4 cells were maintained in IMDM medium supplemented with 15% horse serum and 10% WEHI-3B cell conditioned medium and P/S.

### Construction of plasmids

Murine *Egr-1* promoter fragment (from −1780 bp to + 21 bp) was generated by PCR from BAC plasmid (Clone# RP23-108C3, BACPAC Resources) and inserted into pGL3-basic plasmid. c-Fos promoter (from −1141 bp to + 19 bp) luciferase reporter construct was a generous gift from Dr. Wan-Wan Lin (National Taiwan University). The Dox-inducible Gfi1 expression construct pPMPrtTA-Gfi1-GFP has been described before^19^.

### Mice, bone marrow cell isolation and colony assays

Gfi1 knockout mice^8^ were bred and housed in the animal facility at The University of Toledo. All experiments using mouse BM cells were approved by the Institutional Animal Care and Use Committee (IACUC) of The University of Toledo and were performed per the approved protocol. Bone marrow cells were isolated from 6- to 8-week-old C57BL/6 WT and Gfi1 mutant mice as previously described^18^. Lin^−^ cells were purified using the mouse Lineage Cell Depletion kit (Miltenyi Biotec) and cultured in IMDM media with 10% FBS, 10 ng/ml IL-3, 20 ng/ml IL-6 and 25 ng/ml SCF (Peprotech). For colony forming assay, Gfi1 knockout mice were treated with 5-fluorouracil (50 mg/kg) intraperitoneally prior to isolation of BM cells 5 days later. Cells were cultured in IMDM media containing 10% FBS, 1% P/S, 25 ng/ml SCF, 10 ng/ml IL-3 and 20 ng/ml IL-6 for 1 hour, and then 10^4^ cells were plated in Methylcellulose-based Media (R&D System) containing 10% FBS, IL-3, IL-6, SCF and G-CSF with or without indicated inhibitors. Colonies were counted on day 7.

### Flow cytometry

Cells were first washed in PBS containing 2% horse serum and then blocked using Fc block (eBioscience) for 15 min. Subsequently, cells were incubated with isotype control FITC-conjugated anti-mouse IgG, antibodies against F4/80, Gr-1, Mac-1, Mac-3, G-CSFR or M-CSFR for 30 min prior to washing in PBS with 2% horse serum. Cells were analyzed by two-color flow cytometry on an LSR Fortessa (BD Biosciences) using FACSDiva and analyzed with FlowJo (Tree Star).

### Western blot analysis

The experiments were performed as previously described^18^. Cells were lysed in SDS lysis buffer. Proteins were separated by SDS-PAGE and then transferred onto polyvinylidenedifluoride (PVDF) membranes. The membranes were incubated with the antibodies against phospho-Erk1/2, c-Fos, Egr-1, Egr-2, or β-actin (Cell Signaling), followed by detection of signals using enhanced chemiluminescence.

### Transient transfection and luciferase reporter assay

Cells were transiently transfected by electroporation with the luciferase reporter constructs containing the *c-Fos* or *Egr-1* promoter fragment. After recovering in complete culture medium for 16 hours, cells were washed and placed in RPMI-1640 medium containing 10% FBS and 10 ng/ml G-CSF for 8 hours. Cells were harvested and luciferase activities were measured using luciferase reporter kit and Molecular Devices Lmaxluminometer (Sunnyvale, CA).

### Real-time reverse transcription polymerase chain reaction (qRT-PCR)

Total RNA was extracted using TRIzol reagent (Invitrogen) and reverse transcribed into cDNA using the GoScript™ Reverse Transcription System and Oligo(dT)15 primer (Promega, Madison, WI). The relative mRNA levels of the different genes were quantitated by qRT-PCR using the SsoFast^TM^ EvaGreen Supermix^®^ kit (Bio-Rad) following normalization to GAPDH mRNA expression.

### Apoptosis assay

Apoptosis was examined using the Annexin V-PE apoptosis detection kit (BD Biosciences) as previously described^18^. Briefly, 0.3 × 10^6^ cells were collected and incubated with Annexin V-PE and 7 amino-actinomycin (7-AAD). Cells were analyzed by two-color flow cytometry as described above.

### Chromatin immunoprecipitation assay (ChIP assay)

ChIP assays were performed essentially as described^32^. Briefly, 32D cells were fixed with 1% formaldehyde and then lysed in hypotonic buffer [5 mM Tris-HCl (pH 7.5), 85 mM KCl and 0.5% Nonidet P-40]. After centrifugation at 6000 rpm for 5 min, nuclei were lysed in ChIP lysis buffer [1% SDS, 10 mM EDTA, and 50 mMTris HCl (pH 7.5)] and sonicated to shear chromatin DNA to ~500-bp fragments. Nuclear lysates were precleared with protein A/G agarose beads and rabbit normal IgG for 1 h and subjected to immunoprecipitation using the anti Gfi1 or a species-matched irrelevant antibody. Precipitated DNA was examined by semi-quantitative PCR.

### Statistics

Statistical analyses were performed using GraphPad Prism software (GraphPad Software, La Jolla, CA, USA). Data are presented as mean ± SD in the figures. A p value < 0.05 was considered significant and shown as * with P < 0.01 shown as ** and P < 0.001 as ***.

## Supplementary information

Supplementary Information

## Data Availability

No datasets were generated or analysed during the current study.
