# Supplementary material for: Gfi1-Mediated Repression of c-Fos, Egr-1 and Egr-2, and Inhibition of ERK1/2 Signaling Contribute to the Role of Gfi1 in Granulopoiesis
Source: Sci Rep. 2019 Jan 24;9:737. doi: 10.1038/s41598-018-37402-z (PMC6345849; doi:10.1038/s41598-018-37402-z)
Supplement: Supplementary file 1 — Supplementary Information [file 41598_2018_37402_MOESM1_ESM.pdf]

Gfi1-Mediated Repression of *c-Fos*, *Egr-1* and *Egr-2*, and Inhibition of ERK1/2 Signaling Contribute to the Role of Gfi1 in Granulopoiesis

Yangyang Zhang[1], Nan Hu[1] and Fan Dong[2]

Department of Biological Sciences, University of Toledo, Toledo, Ohio 43606

[1] Yangyang Zhang and Nan Hu contributed equally to this work.

[2] Correspondence and requests for materials should be addressed to F.D. (email: fan.dong@utoledo.edu)

Supplementary Figures Legends

Supplementary figure S1. Effects of Gfi1 overexpression on the expression of neutrophil vs monocyte differentiation markers. 32D/Y729F/Gfi1 (**A**) and FDCP/Y729F/Gfi1 (**B**) cells were cultured in G-CSF in the absence (Ctr) or presence of Dox for 6 and 2 days, respectively. The mRNA levels of neutrophil elastase (NE), lactoferin (LF), M-CSF and MMP-12 were examined by qRT-PCR.

Supplementary figure S2. Flow cytometric analyses of cell surface levels of G-CSFR and M-CSFR in unpurified and Lin$^-$ BM cells from *Gfi1*$^{+/+}$ and *Gfi1*$^{-/-}$ mice. BM cells were obtained from 8-week old *Gfi1*$^{+/+}$ and *Gfi1*$^{-/-}$ mice prior to evaluation of G-CSFR and M-CSFR expression.

Supplementary figure S3. Effects of the Mek1/2 inhibitors on the expression of neutrophil vs monocyte differentiation markers in *Gfi1*$^{-/-}$ BM cells. Lin$^-$ cells from *Gfi1*$^{-/-}$ mice were cultured in the absence or presence of U0126 (U0) or PD0325901 (PD) for 3 day. The mRNA levels of myeloperoxidase (MPO), NE, LF, M-CSF and MMP-12 were examined by qRT-PCR.

Supplementary figure S4. Shown is the full-length blot for figure 5A, right panels.

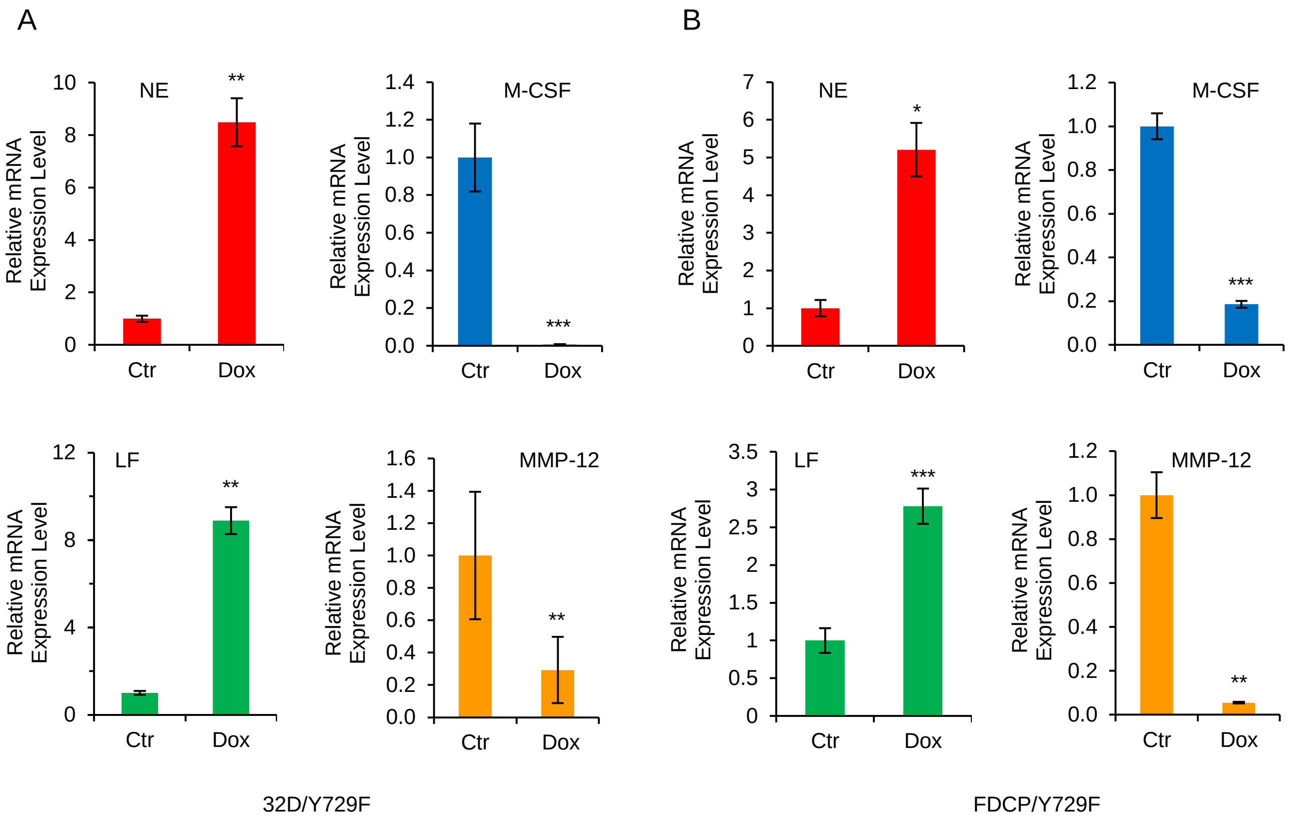

Supplementary figure S1

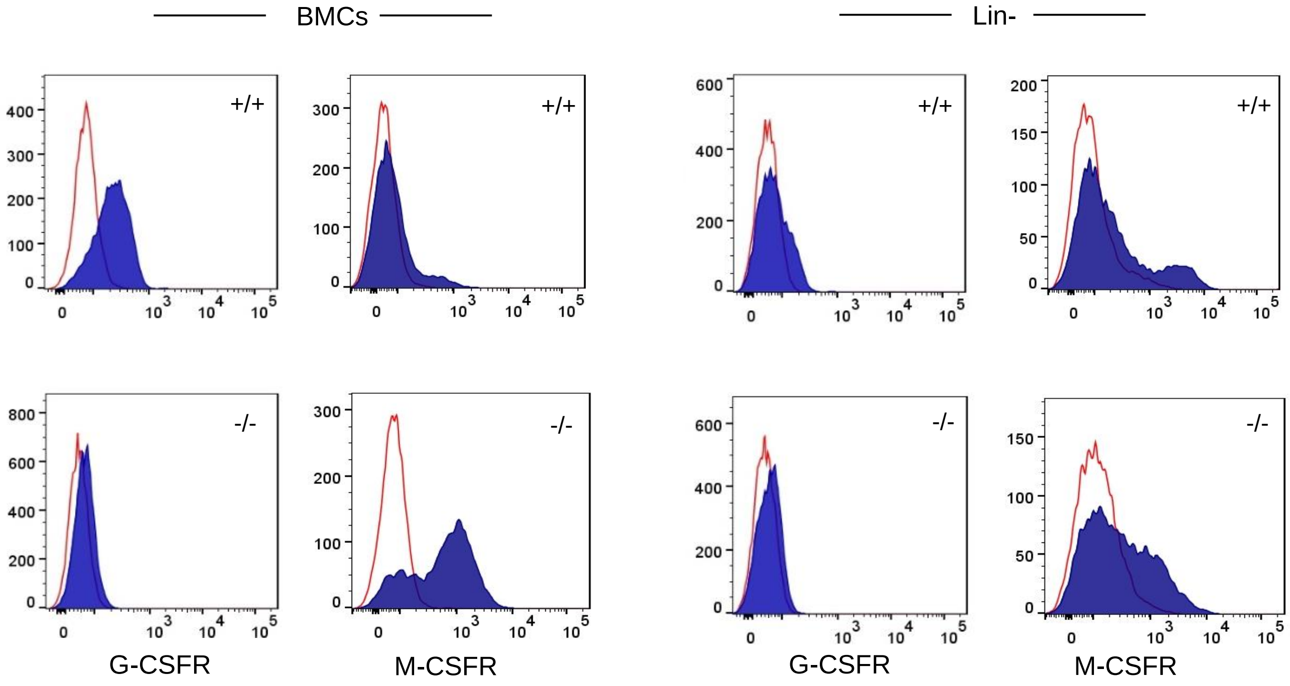

Supplementary figure S2

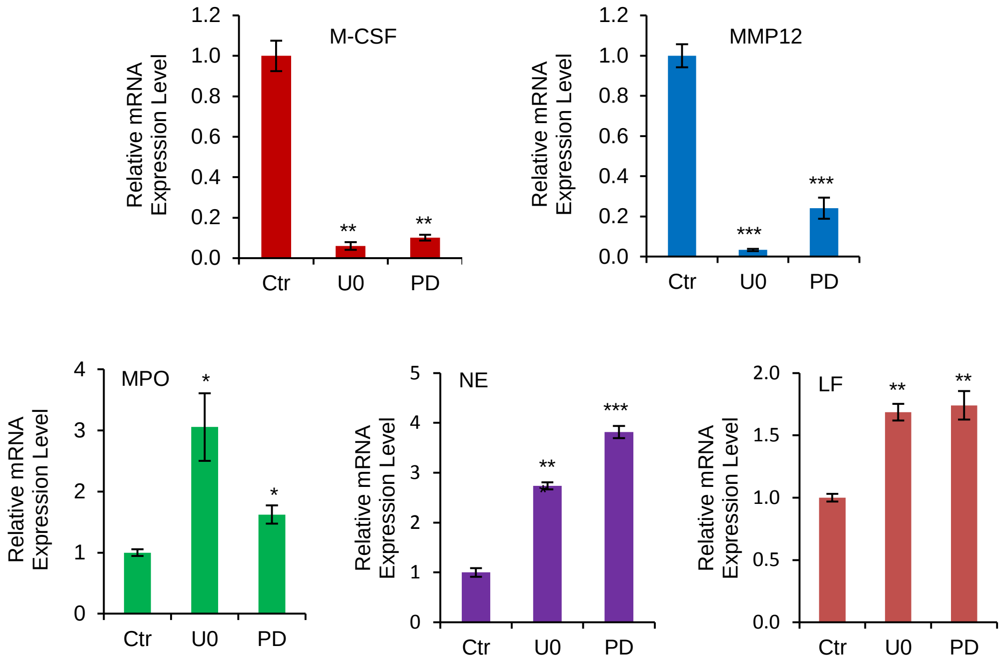

Supplementary figure S3

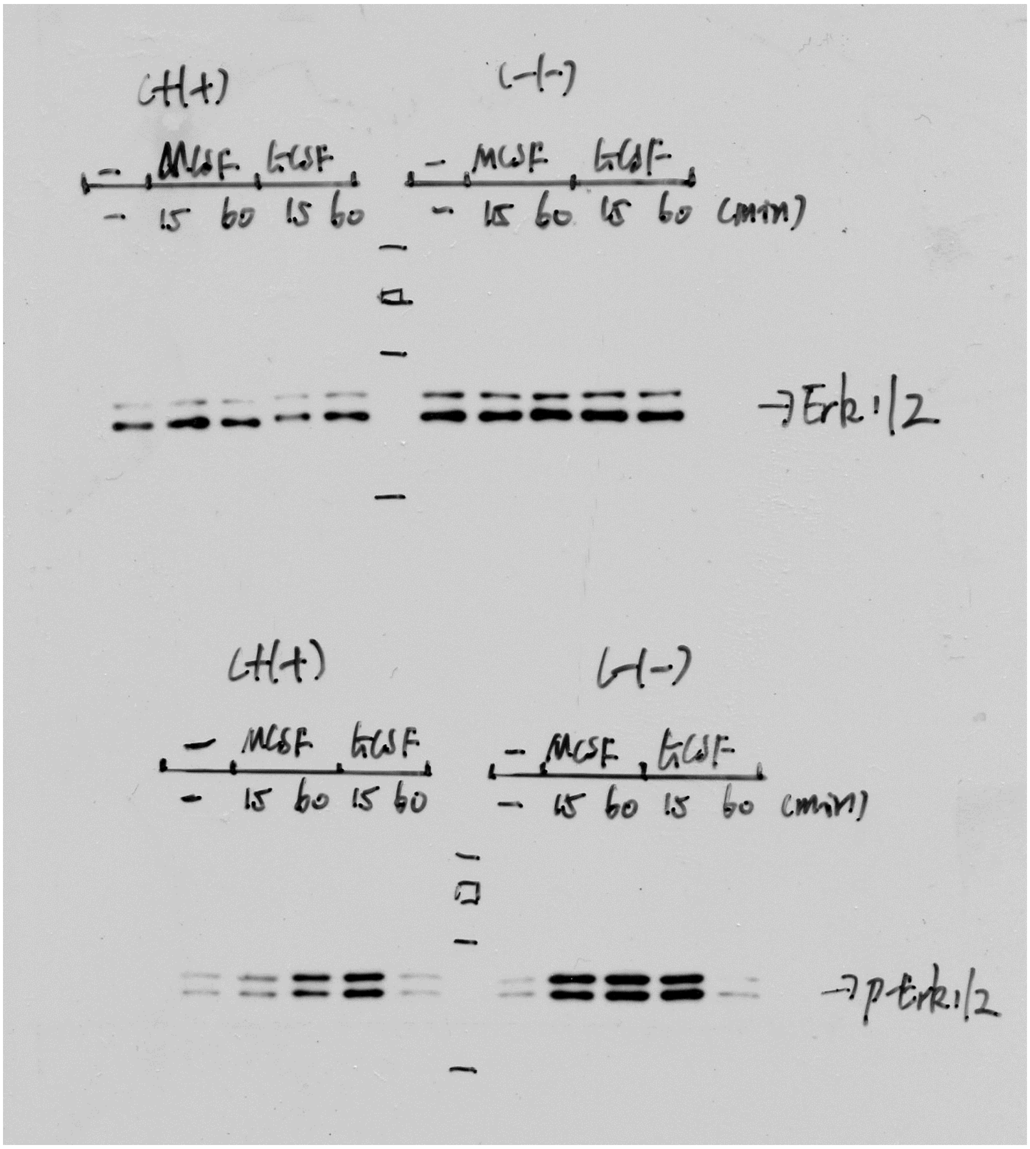

Supplementary figure S4
